# Challenges For Percutaneous Left Atrial Appendage Closure: Imaging And Residual Flow

**DOI:** 10.5935/abc.20180135

**Published:** 2018-07

**Authors:** Tarik Yildirim, Ibrahim Altun, Mustafa Ozcan Soylu

**Affiliations:** Faculty of Medicine, Department of Cardiology Mugla Sitki Koçman Üniversity Tip Fakültesi Orhaniye Mah. Haluk, Mugla – Turkey

**Keywords:** Atrial Appendage, Septal Occluder Devices, Echocardiography, Three-Dimensional, Medical Records

We have read with great interest the paper entitled ‘First results of the Brazilian Registry of Percutaneous Left Atrial Appendage Closure’ by Guérios et al^1^. is a very important study. We have some suggestions about this trial.

Firstly, two-dimensional transeosophageal echocardiography (2D-TEE) can evaluate the morphology of left atrial appendage in multiple views. But real-time three-dimensional transeosophageal echocardiography (3D-TEE) provides more detailed images of the left atrial appendage (LAA) anatomy than 2D-TEE.^2^ A competent physician can accurately evaluate the LAA depth and landing zone. Moreover LAA closure depends on an accurate determination of anatomical structure. Therefore the 3D-TEE may show advantantages in relation to 2D-TEE in transcathater LAA closure.

Secondly, we wonder which indicators were used the device selection. For example, Lefort occluder devices are appropriate for single-lobe appendage, but LAmbre occluder can be used in LAA depth < 21 mm.^2^

Lastly we would like to know about the two patients whose devices showed thrombus formation, as incomplete LAA closure may be associated with an increased risk of thrombus formation and then the second device may be inserted.^3^ Did the authors do further investigation and intervention in these patients?
