# Supplementary material for: Challenges For Percutaneous Left Atrial Appendage Closure: Imaging And Residual Flow
Source: Arq Bras Cardiol. 2018 Jul;111(1):117. doi: 10.5935/abc.20180135 (PMC6078370; doi:10.5935/abc.20180135)
Supplement: Related texts

# Reply

Thank you for your interest in our paper and the pertinent comments. We agree that in comparison to 2D, 3D-TEE provides far more information, security and predictability to percutaneous left atrial appendage closure. In our Service, real-time 3D-TEE is the default tool used for guidance in these procedures. However, the Brazilian Registry of Percutaneous Left Atrial Appendage Closure is a multicenter Registry, which data has been collected since 2010. At that time, and even today, not all centers involved in the Registry had 3D-TEE available in the cath lab, and in those cases, guidance to the procedure was based solely on 2D-TEE information.

Device selection was restricted due to the limited device availability in the Brazilian market. Only the Amplatzer Cardiac Plug (ACP, St Jude Medical, St Paul, MN) was available for use in Brazil until mid-2015, when the Watchman device (Boston Scientific, Marlborough, MA) came into national market. We still do not have the Amulet device (St Jude Medical, St Paul, MN) in Brazil, and LAmbre (Lifetech Scientific, Shenzhen, China) became available in our market only in January 2018.

Regarding the 2 patients in whom thrombus formation was detected at follow-up, in both cases the LAA was completely closed. Thrombus formation was not related to residual LAA flow - hence there was no indication for further procedure – but developed over the surface of the device. Both patients were treated with reinstitution of oral anticoagulation for 3 months with thrombus resolution, and there were no other adverse clinical consequences.

**Ênio E. Guérios, on behalf of the authors of the Brazilian
Registry of Percutaneous Left Atrial Appendage Closure**
